# Secretory Proteins Are Involved in the Parasitism of Melon by *Phelipanche aegyptiaca* During the Attachment Stage

**DOI:** 10.3390/plants13213083

**Published:** 2024-11-01

**Authors:** Lifeng Xiao, Qiuyue Zhao, Xiaolei Cao, Zhaoqun Yao, Sifeng Zhao

**Affiliations:** 1 Key Laboratory of Molecular Breeding and Variety Creation of Horticultural Plants for Mountain Features in Guizhou Province, Kaili University, Kaili 556000, China; 13139933905@163.com; 2 Key Laboratory of Oasis Agricultural Pest Management and Plant Protection Resources Utilization, Shihezi University, Shihezi 832003, China; zhaoqiuyue1@stu.shzu.edu.cn (Q.Z.); tulanduocxl@sina.com (X.C.); yaozhaoqun@shzu.edu.cn (Z.Y.)

**Keywords:** RNA sequencing, *Phelipanche aegyptiaca*, gene expression, HIGs, effector proteins

## Abstract

Parasitic plants represent a significant challenge in global agriculture, with Broomrape (*Orobanche*/*Phelipanche* spp.) being a notable example of a holoparasitic species that targets the roots of host plants. This study employed comparative transcriptomics to investigate the mechanisms underlying the parasitism of *P. aegyptiaca* on melon, focusing on both resistant and susceptible interactions. The findings indicate that the critical phase of *P. aegyptiaca* parasitism occurs during the post-attachment stage. It is suggested that peptidases may play a role in the development of invasive cells, while cell wall-degrading enzymes (CWDEs) are likely involved in cell wall modification and degradation, and transferases, elicitors, and effectors may play a role in immune regulation. In this study, 25 tobacco rattle virus (TRV) recombinant vectors were successfully constructed and functionally validated using a host-induced gene silencing assay to explore the functions of candidate-secreted effector proteins. The results revealed that silencing *Cluster-107894.0*, *Cluster-11592.0*, and *Cluster-12482.0* significantly decreased the parasitism rate of *P. aegyptiaca* on *Nicotiana benthamiana*. Notably, *Cluster-107849.0* encodes a cellulase with hydrolase activity, *Cluster-11592.0* encodes a periodic-dependent kinase inhibitor with phosphoprotein activity, and *Cluster-12482.0* encodes a glucan 1,3-β-glucosidase with hydrolase activity. These findings potentially offer a novel theoretical framework and justification for understanding host–parasite plant interactions, and suggest new avenues for developing crop varieties resistant to parasitic infestation.

## 1. Introduction

*Phelipanche aegyptiaca* belongs to *Orobanche*/Phelipanche spp. in the Orobanchaceae. It is a holoparasitic plant that parasitizes the roots of its host plants [1]. *P. aegyptiaca* has been widely distributed throughout the world, posing a serious threat to agricultural production. It is highly adaptable and can grow in many environments, and is particularly harmful under adverse conditions [2].

Orobanche has a wide host range and has been reported to parasitize about 50 host plant species in 17 families, and is particularly harmful to crop species in the Cucurbit and Solanaceae families [3,4]. Orobanche has a complex life history, with damage to host plants beginning during the underground growth and development period, and it has already caused irreparable damage and harm to host plants by the time it emerges from the ground [5,6], making Orobanche eradication a worldwide challenge.

The processes and mechanisms by which parasitic plants harm host plants include the following: seeds under suitable conditions absorbing water and swelling, and germinating after sensing germination stimulants secreted by the host [7]; forming haustorium induced by host-secreted haustorium-inducing factors [8]; haustorium coming into contact with the host root and forming intrusive cells after the contact with the host root [9]; further establishing a connection with the host through the formation of a xylem bridge [9]; taking up water and nutrients from the host [10,11]; and molecular communication with the host plant [12,13,14].

Transcriptome sequencing has been widely used in the study of parasite–host plant interaction mechanisms [15,16], such as sunflower with *Orobanche cumana* [17,18,19,20], faba bean with *Striga* [21], and tomato with *Cuscuta* [22,23]. Genetic and molecular information is severely lacking in *P. aegyptiaca*, and transcriptome sequencing allows for the analysis of any species without reference to genetic information. Parasitism-related genes of *Orobanche* have also been studied through transcriptome sequencing. For example, analysis results of differentially expressed genes (DEGs) in different parasitism stages of several Orobancheaceae plants showed that the expression of genes encoding cell wall-modifying enzymes was generally up-regulated [24,25]. The invading cells grow towards the host vascular bundles under mechanical pressure and cell wall-modifying enzymes, after haustorium cells of parasitic plants enter the host cortex [25].

All strategies for pathogen infection of plants require the release of effectors into host plant cells to initiate successful infection and colonization [26], manipulate host gene expression, disrupt host cell wall barriers, or interfere with host recognition [27]. Similar to phytopathogenic bacteria, the invasion of a host by a parasitic plant requires the secretion of effector proteins that promote effective invasion by manipulating the host. One study identified parasitism-related genes were up-regulated during haustorium development after attachment to the host in three Orobanchaceae parasitic species [28], where genes encoding proteases, CWDEs, and extracellular secretory proteins were highly up-regulated. Similarly, transcriptome analysis of *Cuscuta* showed increased expression of genes encoding CWDEs during the infection phase [29]. Transcriptome assemblies used to identify *Striga* and *Orobanche* genes were used to study the involvement of virulence proteins on a genome-wide scale [30,31]. The evolution of parasitic plant virulence effectors has also been identified, with several different *S. hermonthica* transcripts identified based on whether *S. hermonthica* grows on maize or sorghum [32], and these differential transcripts include genes that are involved in both defense mechanisms and pathogenesis, and may be parasitic plant effectors. High transcript levels have been used as a filter for pathogen identification with effector molecules [33], and differences in gene expression have been suggested to be responsible for differences in virulence [34]. The sensitivity of high-throughput comparative transcriptomics offers the possibility of identifying and screening potential candidate effector proteins of *P. aegyptiaca*.

Melon is one of the most widely grown and economically profitable fruit crops in the world [35]. However, as one of the main hosts of *P. aegyptiaca*, the parasitic damage has seriously threatened the development of melon. Selecting resistant varieties is the most cost-effective technology and method from the perspective of the long-term goal of sustainable agricultural development [6]. For the development of genetic resistance strategies, it is crucial to fully understand the parasitism mechanism of the parasitic plants.

Building upon the findings of a prior evaluation concerning the resistance of melon varieties to *P. aegyptiaca* parasitism [36], the melon variety “KR1326”, identified as resistant to *P. aegyptiaca*, and the variety “K1237”, identified as susceptible, were selected as subjects for this study. The interactions between *P. aegyptiaca* and melon were simulated utilizing both the root chamber method and the potting method. Meanwhile, based on the results of screening candidate-secretion effector proteins from the secretome of *P. aegyptiaca* [37], 25 candidate-secretion effector proteins (CSEPs) from *P. aegyptiaca* were functionally verified and analyzed using the host-induced gene silencing (HIGs) assay. This study aims to provide new ideas for analyzing the parasitism mechanism of *P. aegyptiaca* and to provide a theoretical basis for further development of parasitic plant control targets.

## 2. Results

### 2.1. Phenotypic Differences of P. aegyptiaca in Interacting with Different Melon Varieties

The findings from the pot experiment indicated that the growth of the “K1237” cultivar, when inoculated with *P. aegyptiaca*, was substantially inhibited, resulting in significant dwarfism. Additionally, a considerable number of *P. aegyptiaca* plants reached the flowering stage, with a parasitism rate of 100%. In contrast, the growth of the “KR1326” cultivar remained robust, with no emergence of *P. aegyptiaca* plants (Figure S1A,B). The parasitism status of *P. aegyptiaca* on the roots of “KR1326” and “K1237” was observed by washing the roots. It was found that *P. aegyptiaca* seedlings in different growth stages were heavily parasitized on the roots of “K1237”, but only a few *P. aegyptiaca* on the roots of “KR1326” and stagnated at the nodule stage (Figure S1C,D).

**Figure 1:**
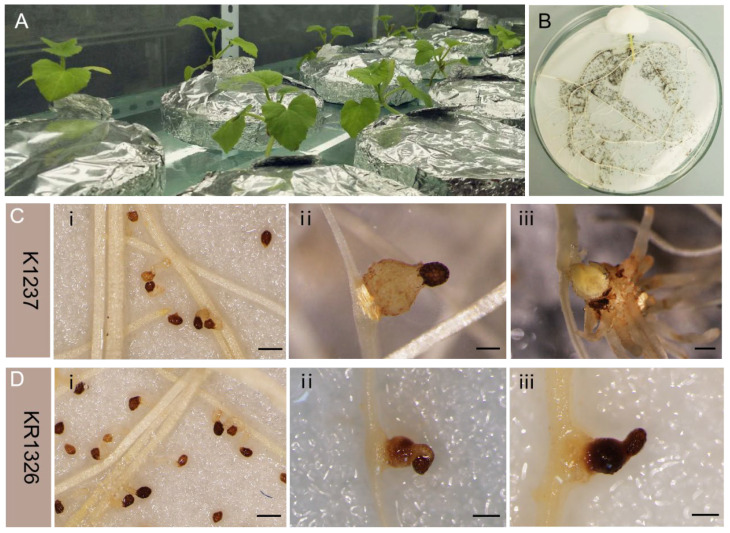
Differential performance of *P. aegyptiaca* in interaction with melon in the root chamber method. (**A**,**B**) The interaction of *P. aegyptiaca* with melon roots. Differential phenotypes of *P. aegyptiaca* in interaction with “KR1326” (**C**) and “K1237” (**D**), including the periods of 7 dpi (i), 14 dpi (ii), and 21 dpi (iii), with a scale of 1 mm.

Significant phenotypic differences were also observed in the root chamber method test, where most of the *P. aegyptiaca* seeds had germinated by 7 dpi (Figure 1Ci,Di). At 14 dpi, *P. aegyptiaca* had successfully established connections with the vascular system of the “K1237” roots, with expanded nodules, an important feature for successful vascular connection formation with the host (Figure 1(Cii)); however, browning and necrosis of *P. aegyptiaca* were more readily visible around the “KR1326” roots (Figure 1(Dii)). By 21 dpi, more *P. aegyptiaca* nodule expansion and cotyledon expansion phenotypes occurred in the *P. aegyptiaca*–K1237 compatible interaction (Figure 1(Ciii)), in contrast to the complete stagnation of *P. aegyptiaca* growth on “KR1326” roots (Figure 1(Diii)).

The validation results indicated that there were significant differences in resistance to *P. aegyptiaca* between “K1237” and “KR1326”, and that the critical period for the establishment of parasitism by *P. aegyptiaca* on melons occurred during the attachment period, which is further subdivided into the pre-attachment and post-attachment periods. Transcriptome samples of *P. aegyptiaca* at the early (9 dpi) and late (16 dpi) stages of attachment were collected and sequenced to further investigate *P. aegyptiaca*’s molecular basis of interaction with melon.

### 2.2. Analysis of DEGs in P. aegyptiaca

The quality assessment and qRT-PCR validation of the transcriptome sequencing results have confirmed the accuracy and reliability of the transcriptome data quality [37]. Further analyses revealed that, in addition to the relatively large number of gene sequence data sets common to each sample, the data set of genes co-specifically expressing L_S2 and L_R2 was also large (Figure S2A). The smallest number of sequence sets was found in L_R1. However, a large number of genes were specifically expressed in L_S1, which was mainly reflected in two parts: the genes up-regulated in *P. aegyptiaca* in susceptible interactions, especially in the pre-attachment stage; and the genes up-regulated in *P. aegyptiaca* in the post-attachment period, especially the genes co-expressed in the resistance/susceptibility interactions (Figure S2A). These results provide important direction for subsequent analyses. The exact number of DECs can be seen in Figure S2B.

#### 2.2.1. KEGG Enrichment Analysis of DEGs

**Figure 2:**
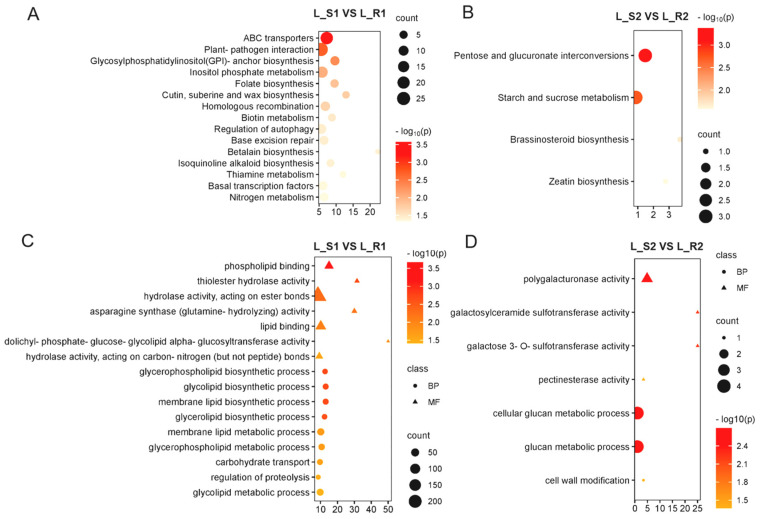
Enrichment analysis of up-regulated genes in *P. aegyptiaca*. Bubble plot of KEGG enrichment of up-regulated genes in *P. aegyptiaca* on “K1237” at 9 dpi (**A**) and 16 dpi (**B**). Pathway associated with cell wall degradation in GO enrichment of up-regulated genes in *P. aegyptiaca* on “K1237” at 9 dpi (**C**) and 16 dpi (**D**).

The DEGs of *P. aegyptiaca* at 9 dpi were enriched in 15 KEGG pathways (Figure 2A), and at 16 dpi were enriched in 4 KEGG pathways (Figure 2B). There were significantly more pathways enriched at 9 dpi, many of which were involved in cell regulation, development, reproduction, and apoptosis, such as inositol phosphate metabolism, folate biosynthesis, biotin metabolism, alkaloid biosynthesis, and nitrogen metabolism, reflecting strong life activities. This indicates that *P. aegyptiaca* inoculated on “K1237” was in a period of rapid growth and development, and enriched for keratin, sialic acid, and wax biosynthesis, associated with cell wall formation. It was initially hypothesized that the critical period for the establishment of the parasitic relationship between *P. aegyptiaca* and melon was the pre-attachment stage.

#### 2.2.2. GO Enrichment Analysis

The results of GO enrichment analysis showed that, at 9 dpi, the up-regulated genes of *P. aegyptiaca* parasitized on “K1237” were enriched to 16 terms related to cell wall degradation (Figure 2C), including seven MF terms: phospholipid binding, thioster hydrolase activity, hydrolase activity, acting on ester bonds, etc., and nine BP terms: glycerophospholipid biosynthetic process, glycolipid biosynthetic process, membrane lipid biosynthetic process, regulation of proteolysis, etc. At 16 dpi, the up-regulated genes were enriched to seven terms related to cell wall degradation (Figure 2D), including four MF terms: polygalacturonase activity, galactosylceramide sulfotransferase activity, galactose 3-O-sulfotransferase activity, and pectinesterase activity, and three BP terms: cellular glucan metabolic process, glucan metabolic process, and cell wall modification. It is hypothesized that *P. aegyptiaca* participates in the modification and degradation of the melon cell wall through CWDEs after attaching to the melon root to break down the physical barrier of the host and promote its successful invasion.

**Figure 3:**
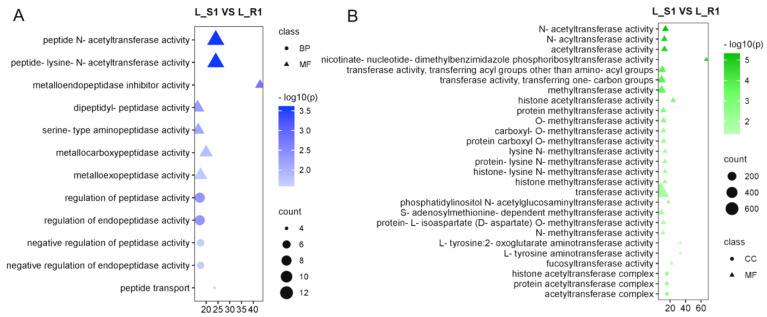
GO enrichment analysis of up-regulated genes in *P. aegyptiaca*. GO pathway enrichment of up-expressed genes in *P. aegyptiaca* on “K1237” associated with peptidase activity-related pathway (**A**) and transferase activity-related pathway (**B**) at 9 dpi.

Further analysis of *P. aegyptiaca*’s DEGs in the susceptible interaction at 9 dpi revealed that many of the GO terms enriched were related to peptidase and transferase activities. The terms enriched for peptidase activity (Figure 3A) included seven MF terms, which were related to acetyltransferase activity and peptidase activity, and five BP terms, which were related to the regulation of peptidase activity. GO terms enriched for transferase activity (Figure 3B) contained 24 MF terms and 3 CC terms related to acyltransferases, methyltransferases, aminotransferases, and other transferase classes. The abundance of peptidase activity and transferase activity may be indicative of physiological and biochemical responses specific to *P. aegyptiaca* during host invasion, such as the regulation of host immune responses.

Analyses of DEGs for different inoculation periods showed that the up-regulated genes of *P. aegyptiaca* parasitized on “K1237” and “KR1326” at 16 dpi were both enriched in genes related to cell wall degradation-related GO terms (Figure S3), suggesting that a few individual *P. aegyptiaca* in the resistance interaction broke through host immunity at the late stage of attachment, and showed significant activities of modification and degradation of the host cell wall and attempted to establish connections with melons, but these responses appeared to be delayed significantly.

#### 2.2.3. Pathogenesis Pathway Analysis

**Figure 4:**
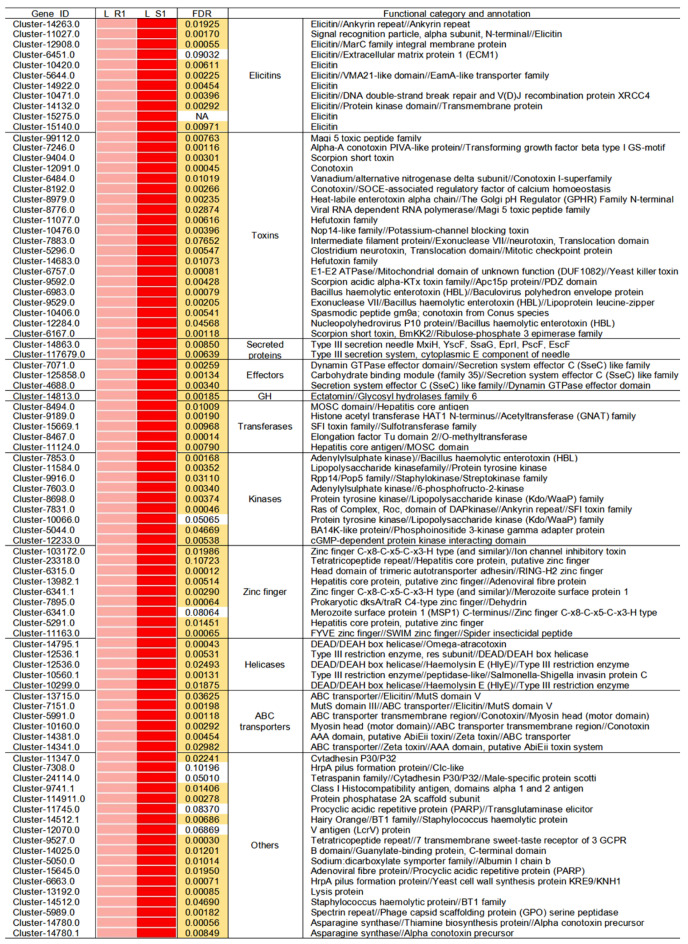
Analysis of up-regulated genes in *P. aegyptiaca* enriched in the pathogenesis pathway at 9 dpi. Columns 2 and 3 indicate the log_2_(fold change) level of gene expression, and the color intensity correlates with the change level. Sample group names are shown at the top.

It was initially determined that the pre-attachment period was a critical period for *P. aegyptiaca* parasitism, so the DEGs were further analyzed. It was found that the up-regulated genes of *P. aegyptiaca* interacting with “K1237” were enriched into the pathogenesis pathway (Figure 4), and the functional annotation results found that 11 genes were annotated to Elicitin and 20 genes were annotated to Toxin. Two genes were annotated to the Type III secretion system; three genes were annotated to the Secretion system effector C-like family; one gene was annotated to Glycosyl hydrolases family 6; five genes were annotated to Transferase; nine genes were annotated to Kinase; nine genes were annotated to Zinc finger; five genes were annotated to Helicase; six genes were annotated to ABC transporter. Genes were annotated to ABC transporter and 18 genes were annotated to other functional information.

**Figure 5:**
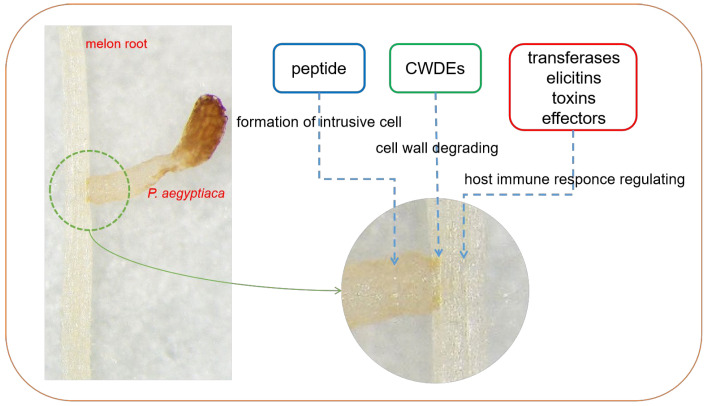
Susceptibility interactions established by *P. aegyptiaca* with melon roots.

Based on the results of the comparative analysis of the *P. aegyptiaca* transcriptome, it was hypothesized that peptidases are involved in the formation of invading cells, CWDEs are involved in the modification and degradation of the melon cell wall to facilitate the invasion, and transferases, excitins, and effectors, among others, are involved in the immune modulation of the host melon to ensure successful establishment of the parasitism relationship (Figure 5).

### 2.3. Results of HIGs Validation

**Figure 6:**
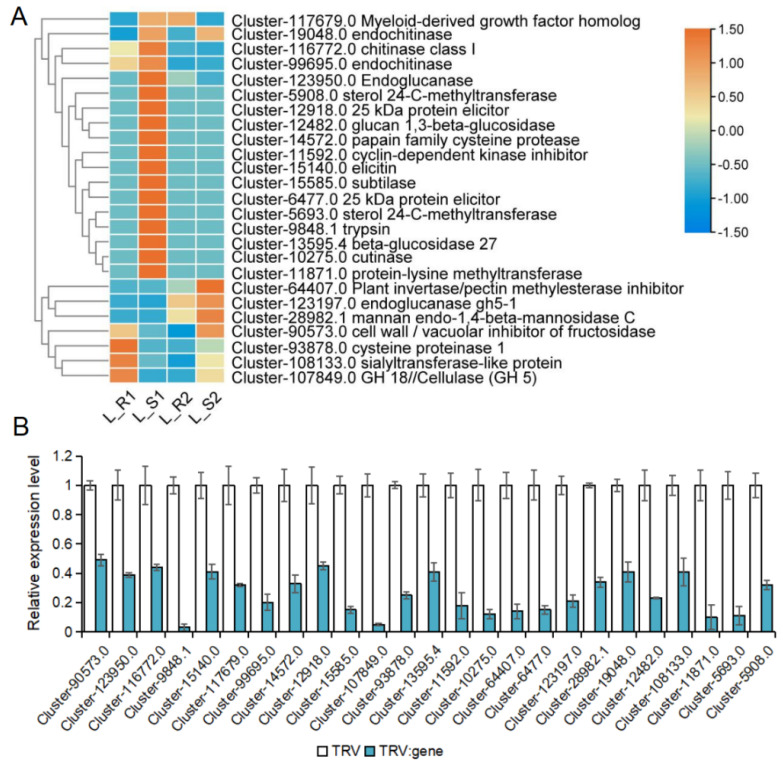
Analysis and validation of 25 candidate-secreted protein genes of *P. aegyptiaca* in HIGs assay. (**A**) Heatmap of the 25 candidate genes with functional annotation information for which recombinant vectors were successfully constructed (**B**), qRT-PCR verification. Relative expression was normalized to endogenous control *Patublin1*. The data shown are the means ± SD of three technical replicates.

Target fragments of 25 CSEP gene sequences of *P. aegyptiaca* were successfully amplified and constructed recombinantly with the TRV2 vector, which encodes growth factor, chitinase, cellulase, glucanase, glucosidase, protein exciters, Bacillus subtilis proteases, cysteine proteases, protease repressors, transferases, and other proteins (Figure 6A). The effect of silencing of *P. aegyptiaca* CSEPs on the parasitism rate was verified by this study. TRV2 empty vector and PDS-containing TRV2 were used as negative and positive controls, respectively, for the HIGs assay in *N. benthamiana*. The qRT-PCR validation results showed that the gene silencing efficiency could reach as low as 51% (Figure 6B).

**Figure 7:**
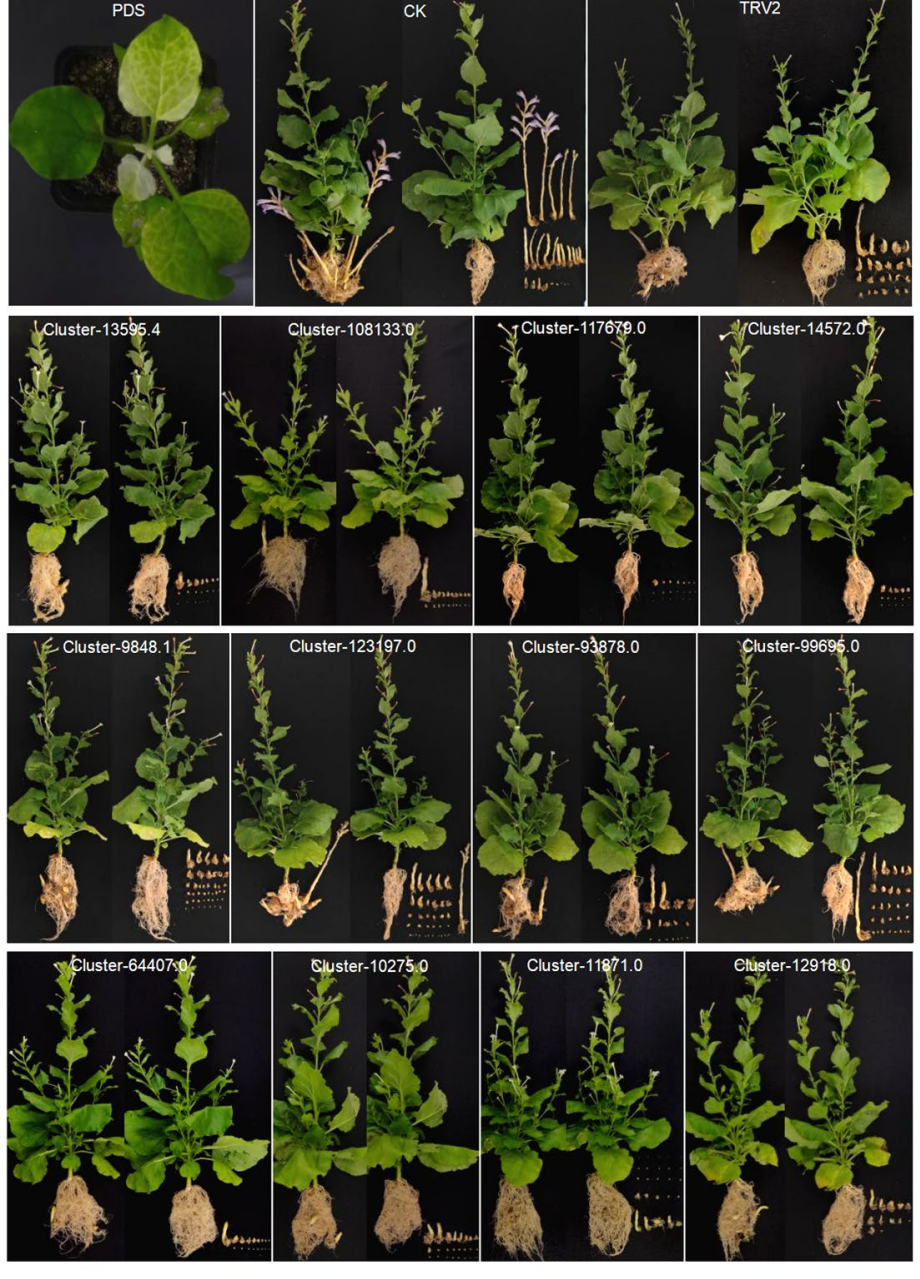
Plant phenotypes in HIGs validation. “PDS” shows the albinism of *N. benthamiana* after expressing TRV: PDS. “CK” was the blank control, and *N. benthamiana* was only treated with *P. aegyptiaca* inoculation. “TRV2” was the *N. benthamiana* expressed empty TRV vector, as a negative control. Transformed *N. benthamiana* expressing the TRV: gene after root washing (**left**), as well as a detailed display of *P. aegyptiaca* (**right**).

**Figure 8:**
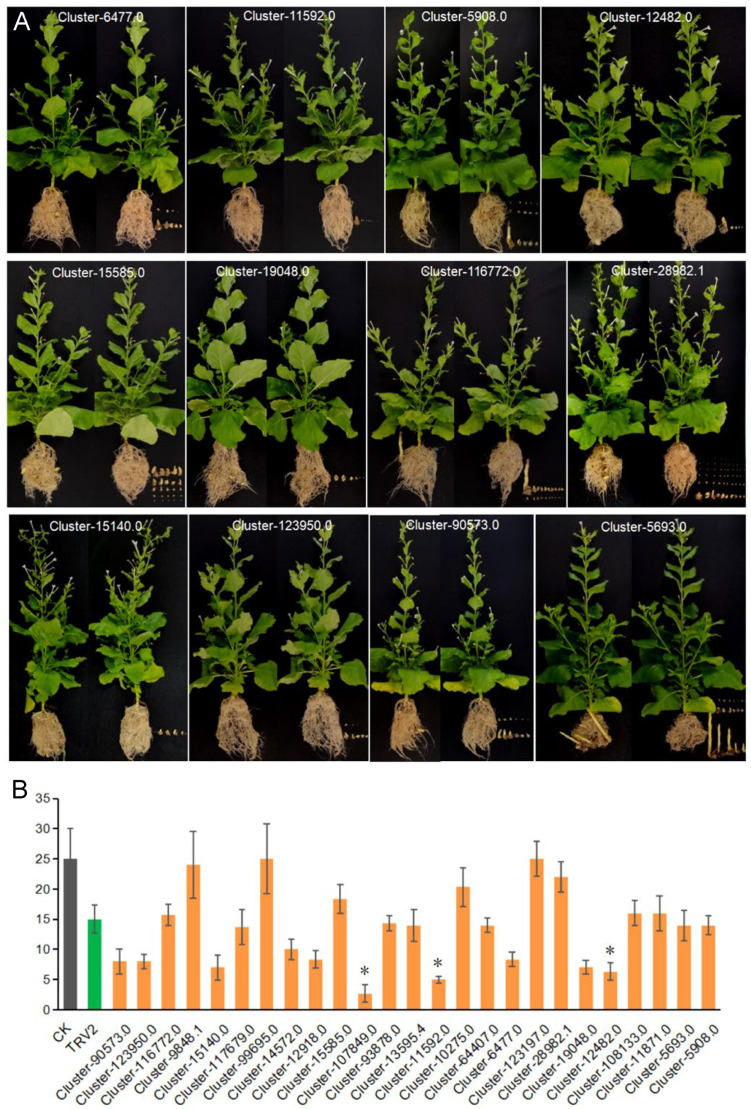
Plant phenotypes in HIGs validation and the quantity statistics. (**A**) Transformed *N. benthamiana* expressing the TRV: gene after root washing (**left**), as well as a detailed display of *P. aegyptiaca* (**right**). (**B**), The statistical analysis of the number of *P. aegyptiaca* parasitized. The experiment was repeated 3 times with 6 plants per treatment. “*” indicates *p* < 0.05.

*N. benthamiana* seedlings injected with TRV: PDS would develop albinism in new leaves at around 7 days (Figure 7). Root washing of *P. aegyptiaca*-inoculated *N. benthamiana* plants two months later showed that *P. aegyptiaca* parasitism on some *N. benthamiana* plants expressing the TRV: gene was significantly reduced compared to the positive control “TRV2”, including silencing *Cluster-90573.0*, *Cluster-6477.0*, *Cluster-123950.0*, *Cluster-15140.0*, *Cluster-12918.0*, *Cluster-11592.0*, *Cluster-107849.0*, *Cluster-19048.0*, and *Cluster-12482.0* genes of transformed *N. benthamiana* (Figure 7 and Figure 8A). Further, the number of *P. aegyptiaca* parasitized in the root system of *N. benthamiana* was counted and analyzed, and it was found that the *P. aegyptiaca* parasitism of *N. benthamiana* plants silenced with genes *Cluster-107849.0*, *Cluster-11592.0*, and *Cluster-12482.0* differed significantly from control plants (Figure 8B), especially plants expressing TRV: *Cluster-107849.0* (Figure S4). Among these, *Cluster-107849.0* encodes a cellulase-like hydrolase with hydrolase activity, *Cluster-11592.0* encodes a periodic-dependent kinase inhibitor with phosphoprotein phosphatase activity, and *Cluster-12482.0* encodes a glucan 1,3-β-glucosidase with hydrolase activity.

### 2.4. Signal Peptide Secretion Function Verification

The secretion function of the signal peptides predicted by *Cluster-107849.0*, *Cluster-11592.0*, and *Cluster-12482.0* was verified using the yeast invertase secretion assay, in which the strains transformed with YTK12 and pSUC2 vectors were used as the negative control, and the strains carrying Avr1b signal peptide were used as the positive control. The results showed that the signal peptides of *Cluster-107849.0*, *Cluster-11592.0*, and *Cluster-12482.0* were able to rescue the defect of the YTK12 sucrose converting enzyme gene and enable the strain to secrete sucrose converting enzyme (Figure S5). Further TTC chromogenic reaction assay showed that the fructosidase SUC2 was secreted into the extracellular structural domain as *Cluster-107849.0*, *Cluster-11592.0*, and *Cluster-12482.0* signal peptide Avr1b, which reduces triamcinolone tetrazolium chloride 2,3,5-TTC to the insoluble red 1,3,5-triamcinolone tetrazolium chloride (Figure S5). The results showed that *Cluster-107849.0*, *Cluster-11592.0*, and *Cluster-12482.0* proteins have potential secretion functions and are typical secretory proteins.

## 3. Discussion

The results of the pot experiment and the root chamber method fully demonstrated the differences in the resistance of two melon varieties to *P. aegyptiaca*, and *P. aegyptiaca* was able to successfully establish a complete and functional linkage with the root vascular system of melon in the susceptible interaction with “K1237”, while in the resistance interaction with “KR1326” almost no such linkage occurred (Figure 1). Pre-parasitism establishment resistance of melon to *P. aegyptiaca* (no significant phenotypic differences were shown) and post-parasitism establishment resistance (*P. aegyptiaca* cannot survive to that extent in “KR1326” root) were therefore ruled out, and it was established that the resistance of melon to *P. aegyptiaca* occurs in the parasitism establishment, or what is termed the attachment period (Figure 1).

Transcriptome sequencing allows functional analysis of the genomes of parasitic plants, and differences in gene expression have been suggested to underlie differences in pathogenicity [34]. The specific parasitism mechanism of *P. aegyptiaca* was resolved at the molecular level based on transcriptome sequencing and analysis in this study. Comparative analysis of the synchronized transcriptome of *P. aegyptiaca* showed that up-regulated genes in the pre-attachment stage of *P. aegyptiaca* in the susceptible interaction were heavily enriched in the KEGG pathway (Figure 2A), revealing the exuberant vitality of *P. aegyptiaca* to invade the host, and suggesting that the pre-attachment stage may be the critical period for *P. aegyptiaca* to invade the host. The up-regulated genes in the post-attachment stage were enriched in several phytohormone signaling pathways (Figure 2B), suggesting that some regulatory hormones, such as zeatin, growth hormone, gibberellin, oleoresinol steroids, and cytokinins, were actively involved in the regulation of *P. aegyptiaca*’s own growth and development as well as the host physiology. This is in line with previous studies reporting that *P. japonicum* (Orobanchaceae) overproduces cytokinin phytohormones in the host to manipulate host physiological functions [38]. Cytokinin biosynthesis gene expression is up-regulated in *P. japonicum* haustorium; the phytohormone moves above the host infestation site, and the transferred cytokinin induces host root hypertrophy, which is common in many parasitic plant infections [38,39,40]. Cytokinin treatment with host plant root secretions or exogenous c/tZR induces haustorium formation and up-regulation of related genes, which increases the rate of attachment to the host root system [41].

*P. aegyptiaca* achieves degradation and modification of the cell wall of the melon root by releasing large quantities of CWDEs, thereby forming a physical connection and facilitating its successful parasitism (Figure 2C,D). This is consistent with the conclusion that parasitic plants obtain water and nutrients from the host vascular system by penetrating the host plant cell wall through mechanical forces and/or CWDEs [42], and that resisting parasitic plant invasion by altering the composition of the cell wall and thus forming physical and biochemical barriers is a defense mechanism adopted by a variety of host plants [22]. Reported cell wall-modifying enzymes of parasitic plants that invade their hosts include pectin lytic enzymes, pectin methyl esterases, cellulases, xyloglucanases, polygalacturonases, and carbohydrate-activating enzymes [24,25,28,43,44]. The enrichment of peptidases and transferase also confirmed the physiological manipulation and immunomodulation of *P. aegyptiaca* in susceptible interactions with “K1237” (Figure 3). In the post-attachment stage, *P. aegyptiaca* continued to degrade and modify the melon cell wall by CWDEs (Figure S3) to overcome the ongoing defense response of melon.

The up-regulated genes of *P. aegyptiaca* in susceptible interactions at the pre-attachment stage were significantly enriched in the pathogenesis pathway, and these genes encoded Elicitin Toxin, Type III secretion system, secretion system effector class C family proteins, Glycosyl hydrolases family 6, Transferase, Kinase, Zinc finger, Helicase, ABC transporter, etc. (Figure 4). This suggests a more pronounced pathogenic activity of *P. aegyptiaca*, mainly in the disruption of the melon cell wall, which transforms the melon root from a pathway into an efficient nutrient source reservoir. The disease-associated proteins interfered with the melon defense system, and a large number of effectors were secreted to participate in this process. Previous transcriptomics studies have also shown that once a connection is established between a parasitic plant and a host plant, the parasite–host relationship will depend on multiple transporters to transfer nutrients from the host [28]. Similarly, the S. gesnerioides transcriptome shows that genes encoding cell wall-modifying enzymes and transporter proteins are strongly induced during the pre-haustorium formation and haustorium infection stages [32]. Genes encoding transporter proteins and regulatory proteins (transcription factors and receptor protein kinases, among others) are co-expressed during the parasitological stage and may also be required for haustorium development and function [28].

*P. aegyptiaca* with silenced genes *Cluster-107849.0*, *Cluster-11592.0*, and *Cluster-12482.0* showed a significant decrease in parasitism (Figure 7 and Figure 8). *Cluster-107849.0* encodes a cellulase-like enzyme with hydrolytic enzyme activity, *Cluster-11592.0* encodes a periodic-dependent kinase inhibitor with phosphoprotein phosphatase activity, and *Cluster-12482.0* encodes a glucan 1,3-β-glucosidase with hydrolase activity, fully demonstrating the involvement of secreted proteins of *P. aegyptiaca* in its parasitism process and manipulating the immune regulatory activities of melon. The regulation of host resistance by effectors has been clearly described in plant–pathogen interactions, and the regulation of host immunity by secreted effector proteins can be extended to parasitic weeds, thus providing new insights into the mechanism of parasitic plant–host plant interactions. For example, up-regulated haustorium genes encoding chytridiomycin-like serine proteases [28] are similar to genes that act as virulence factors in bacterial pathogens [29]. In the root-parasitic weed Striga gesnerioides, SHR4z was found to act as an effector, entering the host cytoplasm to interact with the ubiquitin ligase VuPOB1 and inhibit the host defense response [21,45]. More effector proteins in parasitic plants remain to be identified.

Some secreted proteins that do not possess typical characteristics have also been found to have functional characteristics of effector proteins, suggesting that the screening and validation of effector proteins may not necessarily have to conform to a certain class of constraining characteristics, but should instead focus on their intrinsic potential functionality. The study of parasitic plant-secreted proteins will help to determine the mechanism of parasitic plant–host plant interactions, which is extremely important for the study of breeding parasite-resistant crop varieties.

Identifying the effector proteins of parasitic plants and clarifying their possible sites of action in host cells requires a perfect research system, and it is necessary to detect the interactions between effector proteins and target genes in the host through the yeast two-hybrid system, coimmunoprecipitation, bilobal fluorescence complementation, etc., so as to study the interaction mechanism of the host-plant immune system.

## 4. Materials and Methods

The seeds of *P. aegyptiaca* were collected in bulk from a heavily infected processing tomato field in Jimsar, Xinjiang, China, in 2019 (located at 89°18′ E, 43°99′ N). The seeds (KR1326 and K1237) of melon were collected from the Hami Melon Research Center, Xinjiang Academy of Agricultural Science, Xinjiang, China.

### 4.1. Potting Method

The melon seeds were shaken at room temperature for about two days until they germinated, transferred into 1.5 L plastic pots containing nutrient soil, vermiculite (1:1, *v*/*v*), and *P. aegyptiaca* seeds (0.5 g/kg), and then cultivated in a greenhouse (28 °C, 10,000 Lx of light, and 16 h/d of light). After about 60 d, the melon plants were removed from each pot, their roots and growing *P. aegyptiaca* were carefully cleaned, and the phenotypes were then observed and photographed. The parasitism rate was calculated as follows: Parasitism rate = number of parasitized melon plants/total number of melons × 100%.

### 4.2. Root Chamber Method

Melon seeds shaken to germination were planted in plastic cavity trays containing vermiculite, and seedlings were watered with Hoagland nutrient solution and incubated in a greenhouse (temperature 28 °C, light intensity 10,000 Lx, light duration 16 h/d). Melon seedlings were transferred after the 2nd true leaves grew. A sponge filled with water was placed in a 15 cm diameter Petri dish and covered with two layers of 15 cm diameter filter paper, the roots of melon seedlings were laid flat on the filter paper, the seedlings were fixed with skimmed cotton wool (Figure 1A,B), and the Petri dish was replenished with sufficient water. Tin foil was used to cover the surface of the root chamber to prevent the melon root system from being exposed to direct light, and it was placed on a plant culture rack for incubation. After about one week, sterilized *P. aegyptiaca* seeds (75% anhydrous alcohol treatment for 2 min, 1% NaClO treatment for 20 min, and rinsing with water 3 to 5 times) were inoculated uniformly around the melon root system using a pipette to simulate the interaction process. Water was replenished at the appropriate times, and the phenotypes of *P. aegyptiaca* at different stages of growth and development were observed and recorded.

### 4.3. Transcriptome Sample Preparation and Sequencing

*P. aegyptiaca* was co-cultured with melon using the root chamber method described above, and samples were taken sequentially at 9 d and 16 d after the inoculation. Using scissors sterilized with 75% anhydrous ethanol, the intercropping zone within 1 cm of the *P. aegyptiaca* parasitism site was quickly cut, and the water was blotted out with filter paper, with three replicates for each sample, and a mixture of at least 100 mg from three dishes for each replicate. Samples were quickly frozen in liquid nitrogen and stored at −80 °C.

Sample names: L_R1, L_R2, L_S1, L_S2. “R” stands for resistant melon “KR1326”, and “S” stands for susceptible melon “K1237”. “L_R” represents *P. aegyptiaca* interacting with “KR1326”, and “L_S” represents *P. aegyptiaca* interacting with “K1237”. “1” represents 9 d after inoculation (9 dpi), and “2” represents 16 d after inoculation (16 dpi).

Transcriptome sequencing was performed by Beijing Novogene Technology Co., LTD in China, using the Illumina HiSeq 2500 platform.

### 4.4. Transcript Assembly and Sequence Analysis

After filtering the sequencing data, the clean reads were aligned with the host melon genome, and the annotated melon transcripts were removed, the unannotated reads were identified as *P. aegyptiaca* transcripts and then assembled by Trinity, and the assembled *P. aegyptiaca* unigenes were subjected to RNA-seq correlation analysis and gene expression level analysis.

The resulting sequences were functionally annotated in seven major databases, namely Nt (NCBI nucleotide sequences), Nr (NCBI non-redundant protein sequences), Swiss-Prot (A manually annotated and reviewed protein sequence database), KEGG (Kyoto Encyclopedia of Genes and Genomes), Pfam (Protein family), GO (Gene Ontology), and KOG/COG (KOG: euKaryotic Ortholog Groups; COG: Clusters of Orthologous Groups of proteins).

Gene expression levels were analyzed by FPKM (Fragments Per Kilobase Per Million). Differentially expressed genes (DEGs) were identified using DEseq2 with padj < 0.05 and |log_2_Fold Change| > 1.

### 4.5. KEGG and GO Analysis

KEGG pathway enrichment analysis of DEGs was performed using KOBAS 3.0 (KEGG Orthology-Based Annotation System).

GO enrichment analysis was reflected in GO terms enriched for Biological Process (BP), Cellular Component (CC), and Molecular Function (MF). Functional enrichment analysis of DEGs was performed using cluster Profiler 4.0 R.

### 4.6. Validation of Gene Silencing

Plasmid construction and preparation: The target fragment of the gene for the candidate effector protein of *P. aegyptiaca* was amplified and the PCR product was recombined into the pTRV2 vector by one-step cloning at the XbaI and BamHI cleavage sites. The primers are shown in Table S1.

*Nicotiana benthamiana* seedlings of 3~4 leaves were selected for the experiment. Plasmids TRV1, TRV2 (recombinant plasmid containing tobacco pds gene or target gene), and TRV2 empty vector were transformed by electroshocking with *Agrobacterium tumefaciens* GV3101. Single colonies were picked for culture (5 mL) and then expanded (50 mL), then the bacterial culture was centrifuged at 4000 rpm for 10 min. The recovered organisms were dissolved in bacterial suspension (10 mM MES; 10 mm MgCl_2_; 400 µM acetosyringone AS and ddH_2_O), adjusted to an OD of 0.6 (600 nm), and a mixture of TRV1 and TRV2 bacterial fluids was prepared in a 1:1 ratio before injection, incubated, and cultured for 3 h at room temperature. Then, the Agrobacterium suspensions were injected with a 1 mL needleless syringe. The whole leaf was infested from the abaxial surface, and each treatment was repeated with six plants, with two leaves per plant.

After about 7 days, the *N. benthamiana* leaves injected with the *pds* gene would appear to be whitened, and then the other seedlings would be inoculated with *P. aegyptiaca* and potted. Roots were washed and photographed after 60 days for data counting.

### 4.7. qRT-PCR Validation

After the appearance of leaf whitening according to the above method, *N. benthamiana* from different treatments were cultured by the root chamber method as described in 1.2, and samples were collected for RNA extraction 10 d after inoculation with *P. aegyptiaca*.

Total RNA was extracted from each material sample using the All-Style Gold RNA Extraction Kit (ER301-01), and the integrity of the total RNA was analyzed using 1% agarose gel electrophoresis; the concentration and purity were tested using a Nanodrop ND-2000 (NanoDrop Technologies, USA). Then, the total RNA was reverse-transcribed into cDNA using the PrimeScript Reverse Transcription Kit (AU1-01) sourced from Beijing, China. qRT-PCR was performed on an Applied Biosystems 7500 machine. Reaction system: 2 × PerfectStart Green qPCR SuperMix 10 μL, Passive Reference Dye (50×) 0.4 μL, upstream and downstream quantitative primers (10 μmol/L) 0.4 μL each, ddH_2_O 6.8 μL, cDNA 2 μL, total volume 20 μL. Reaction procedure: 94 °C 30 s; 94 °C 5 s; 60 °C 30 s, 45 cycles. The data were subjected to relative expression calculation using the 2^−ΔΔCT^ method and one-way analysis of variance (ANOVA) by SPSS (IBM SPSS Statistics 19.0, USA) software. All treatments were 3 biological replicates and 3 technical replicates. The primers are shown in Table S2.

### 4.8. Verification of Signal Peptide Secretion Function

Recombinant pSUC2 vector: Predict the signal peptide sequence of the candidate effector protein, amplify the target sequence with high-fidelity enzyme, and recombine the amplified product into the pSUC2 vector by one-step cloning at the EcoRI and XhoI cleavage sites. The primers are shown in Table S3.

The recombinant pSUC2 vector was transformed into *Saccharomyces cerevisiae* strain YTK12 and cultured on CMD-W (tryptophan deficient) medium. Positive clones were cultured on YPRAA medium containing 1% yeast extract, 2% peptone, 2% cotton sugar, and 2 µg/mL antimycin A. YTK12 cells transformed with pSUC2-Avr1bSP were used as a positive control, and the empty vector pSUC2 was used as a negative control. The convertase activity was assayed by monitoring the reduction of triphenyl tetrazolium chloride (TTC) to insoluble red 1,3,5-triphenyl tetrazolium.

## 5. Conclusions

The results of this study demonstrated that the *P. aegyptiaca* parasitism on melon involves the formation of invasive cells and the synthesis of CWDEs during the attachment stage, among other processes. Additionally, the study identified three candidate-secreted effector proteins (*Cluster-107894.0*, *Cluster-11592.0*, and *Cluster-12482.0*) associated with the parasitic mechanism. These findings enhance the understanding of the specific molecular mechanisms underlying parasitism and advance the broader comprehension of parasite–host plant interactions. Furthermore, these results highlight potential genetic targets for the development of resistant host plant varieties.

## Data Availability

The transcriptome data of *P. aegyptiaca* in this study have been uploaded to the SRA database in NCBI: https://www.ncbi.nlm.nih.gov/sra/PRJNA873060 (accessed on 25 August 2022).

## Supplementary Materials

The following supporting information can be downloaded at: https://www.mdpi.com/article/10.3390/plants13213083/s1, Figure S1: Differential phenotypes of melon in the potting method; Figure S2: Pooled visualization analysis and differential expression of the *P. aegyptiaca* transcriptome; Figure S3: GO enrichment analysis of *P. aegyptiaca* up-regulated expressed genes associated with cell wall degradation at the late stage of attachment; Figure S4: The phenotype of six transformed *N. benthamiana* expressing TRV: *Cluster-107849.0* and *P. aegyptiaca* individuals parasitizing the roots in HIGs verification. Figure S5: Functional validation of the *Cluster-107849.0*, *Cluster-11592.0*, and *Cluster-12482.0* protein signal peptide; Table S1: Primers used in HIGs validation of candidate effector genes; Table S2: Primers used in qRT-PCR validation of candidate effector genes; Table S3: Primers used in verification of the secretory function of signal peptide.
