# Supplementary material for: Secretory Proteins Are Involved in the Parasitism of Melon by Phelipanche aegyptiaca During the Attachment Stage
Source: Plants (Basel). 2024 Nov 1;13(21):3083. doi: 10.3390/plants13213083 (PMC11548055; doi:10.3390/plants13213083)
Supplement: Supplementary file 1 [file plants-13-03083-s001.zip › plants-3241556-supplementary.pdf]

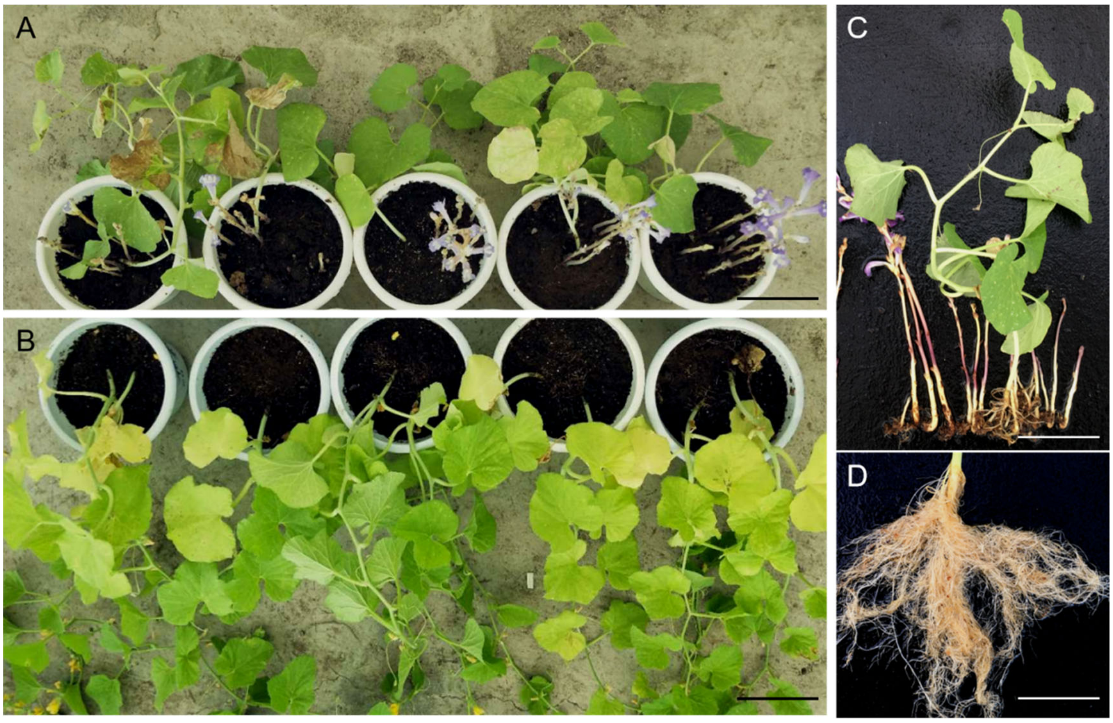

Figure S1. Differential phenotypes of melon in the potting method. A and B show the ‘K1237’ and ‘KR1326’ inoculated with *P. aegyptiaca* under potting conditions, respectively; C and D show the *P. aegyptiaca* grown on the root of ‘K1237’ and ‘KR1326’, with many *P. aegyptiaca* individuals parasitising on the roots of ‘K1237’, and, conversely, no *P. aegyptiaca* individuals that were able to develop fully on the roots of ‘KR1326’. Scale bar is 10 cm.

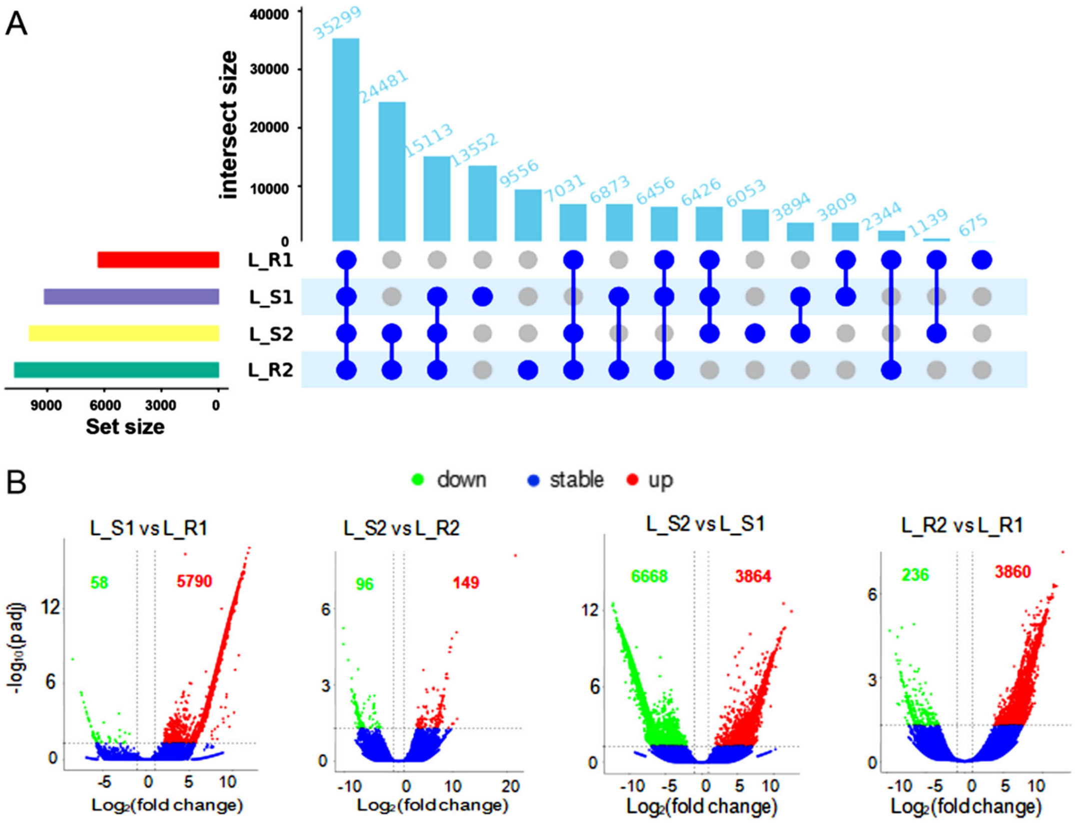

Figure S2. Pooled visualisation analysis and differential expression of the *P. aegyptiaca* transcriptome. A, the horizontal bar in the lower left indicates the size of each quantity set, the dots in the dot plot in the lower right indicate the amount of data unique to a given set of statistics, and the connecting line between the dots indicates the intersection between the data sets, the vertical bar in the upper right indicates the number of quantities corresponding to the unique group or intersection group. B, volcano plot of differentially expressed genes in *P. aegyptiaca*.

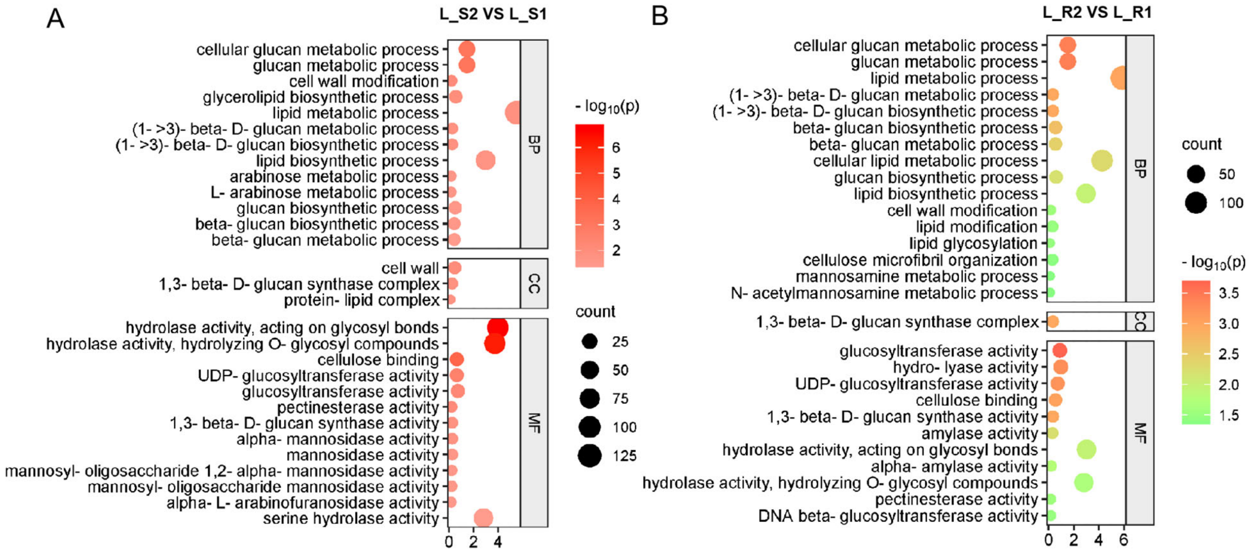

Figure S3. GO enrichment analysis of *P. aegyptiaca* up-regulated expressed genes associated with cell wall degradation at the late stage of attachment. GO enrichment analysis of *P. aegyptiaca* up-expressed genes associated with cell wall degradation on 'K1237' (A) and on 'KR1326' (B) at 16 dpi compared to 9 dpi.

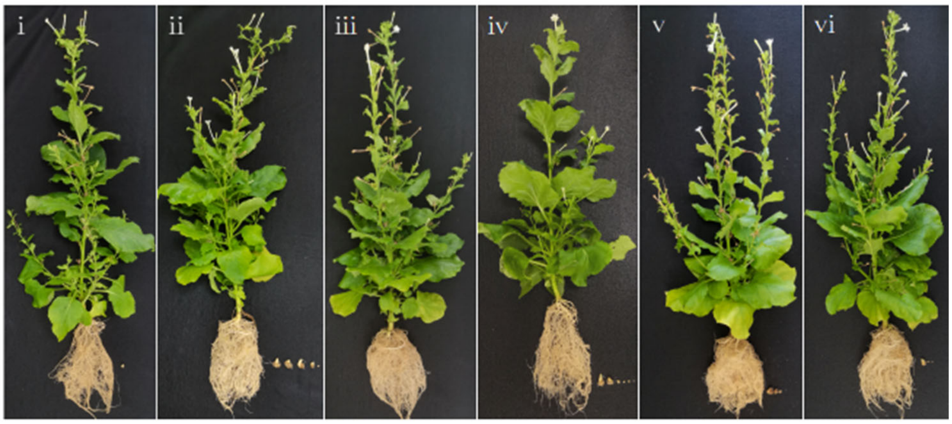

Figure S4: The phenotype of six transformed *N. benthamiana* expressing TRV: *Cluster-107849.0* and *P. aegyptiaca* individuals parasitizing the roots in HIGs verification.

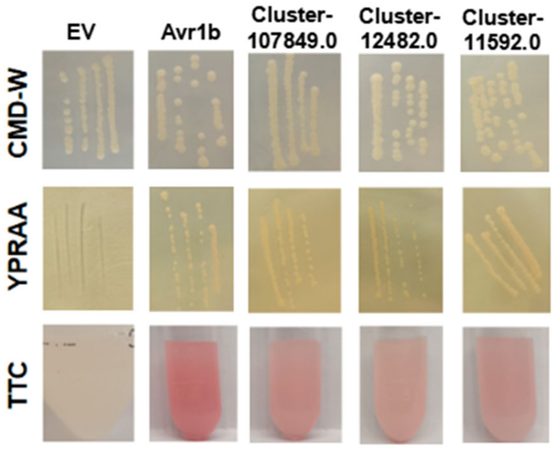

Figure S5: Functional validation of the *Cluster-107849.0*, *Cluster-11592.0*, and *Cluster-12482.0* protein signal peptide. pSUC2-Avr1b was used as a positive control. The pSUC2 vector (EV) was used as negative control. Yeast growth on CMD-W medium confirmed that the vector was transformed into the yeast strain, while growth on YPRAA medium and TTC color change confirmed invertase secretion.

Table S1. Primers used in HIGs validation of candidate effector genes

Gene ID		Sequence（5'-3'）
Cluster-90573.0	F	5'- gtgagtaaggttaccgaattcCTCATAATGGTGGATGCCGTC -3'
	R	5'- cgtgagctcggtaccggatccAAGCTCATGCATATCTTTATTCACACC -3'
Cluster-123950.0	F	5'- gtgagtaaggttaccgaattcAAGGACAACCAAAAAGTGATCTTCG -3'
	R	5'- cgtgagctcggtaccggatccATCAGGGCCAACACAATCGTC -3'
Cluster-116772.0	F	5'- gtgagtaaggttaccgaattcATGCTGAGGAAACTTGGCAAGA -3'
	R	5'- cgtgagctcggtaccggatccGACTTTACCTGCGACGTCCG -3'
Cluster-9848.1	F	5'- gtgagtaaggttaccgaattcACGCGTGCCCAGGGTGCT -3'
	R	5'- cgtgagctcggtaccggatccAAGCGAGTCGATCCAGCTCTT -3'
Cluster-15140.0	F	5'- gtgagtaaggttaccgaattcCAGGCTGAGAGCAGCCCTG -3'
	R	5'- cgtgagctcggtaccggatccAGCGGTCGCAACAACAGTG -3'
Cluster-117679.0	F	5'- gtgagtaaggttaccgaattcGAACACACTGTTGAGAGAGAAGATAGC -3'
	R	5'- cgtgagctcggtaccggatccGATAGCATTAACGATACCTTGGTAATAA -3'
Cluster-99695.0	F	5'- gtgagtaaggttaccgaattcACCTCCCTCTCCACCACCC -3'
	R	5'- cgtgagctcggtaccggatccCTGGCCGAAGGCTTCTGC -3'
Cluster-14572.0	F	5'- gtgagtaaggttaccgaattcAACCCAGATGCCAACTTCTCC -3'
	R	5'- cgtgagctcggtaccggatccCGATGGGAACATACCACCATTG -3'
Cluster-12918.0	F	5'- gtgagtaaggttaccgaattcGCCGCATCGGCCAAGGTC -3'
	R	5'- cgtgagctcggtaccggatccCTTGAAGAACGCCTTCGCG -3'
Cluster-15585.0	F	5'- gtgagtaaggttaccgaattcAACCTCATCGTTACGATGAAGCA -3'
	R	5'- cgtgagctcggtaccggatccCAGACGAAGCTCCTCACGGA -3'
Cluster-107849.0	F	5'- gtgagtaaggttaccgaattcCCAAGAAGACGTACAACAAAAATCA -3'
	R	5'- cgtgagctcggtaccggatccAGTGGAAACCATAATAGAGCAACCA -3'
Cluster-93878.0	F	5'- gtgagtaaggttaccgaattcCTCAACAAAAGATTCTCCATTTTCC -3'
	R	5'- cgtgagctcggtaccggatccAGCCCAGCATGATCCACATT -3'
Cluster-13595.4	F	5'- gtgagtaaggttaccgaattcACGAAGATGTACGAATGCTCGA -3'
	R	5'- cgtgagctcggtaccggatccCTTCGACTCCGCATCACTGAA -3'
Cluster-11592.0	F	5'- gtgagtaaggttaccgaattcGCCCAAGGTTTCGACTGGA -3'
	R	5'- cgtgagctcggtaccggatccAGCAGGCGTAGGAGCAGGC -3'
Cluster-10275.0	F	5'- gtgagtaaggttaccgaattcAACCCTCTGAAGCTGTTTGGTC -3'
	R	5'- cgtgagctcggtaccggatccAAAGCTTGGGAACTTGAAGCC -3'
Cluster-64407.0	F	5'- gtgagtaaggttaccgaattcGCTTACATAGTCTTCAGAAAAGCGTC -3'
	R	5'- cgtgagctcggtaccggatccGACCGCTACAGCTTGACAAATACT -3'
Cluster-6477.0	F	5'- gtgagtaaggttaccgaattcAAGGACTCGCCGCTCACAG -3'
	R	5'- cgtgagctcggtaccggatccGTCCGTGCGCTCGAGAGC -3'
Cluster-123197.0	F	5'- gtgagtaaggttaccgaattcGCTCCTCCTCCTCCTGCTTC -3'
	R	5'- cgtgagctcggtaccggatccCAGGCTGGTCTGGGTGAGACG -3'
Cluster-28982.1	F	5'- gtgagtaaggttaccgaattcGCTGGAAGCAACGCCTACTTC -3'
	R	5'- cgtgagctcggtaccggatccGGTCAGTGCGAGGACAAGCTT -3'
Cluster-19048.0	F	5'- gtgagtaaggttaccgaattcTGGTGCGACACCTCCGCC -3'
	R	5'- cgtgagctcggtaccggatccCTGCGCCTGCTCGCTGTT -3'
Cluster-12482.0	F	5'- gtgagtaaggttaccgaattcTGGGCGCTCCAGGGAGAC -3'
	R	5'- cgtgagctcggtaccggatccCCGGTAGATGGCCTCACTTCC -3'
Cluster-108133.0	F	5'- gtgagtaaggttaccgaattcACATGGGAACAGGTGCGAAA -3'
	R	5'- cgtgagctcggtaccggatccAGGTGCACCATTCTCTGTGACA -3'

Cluster-11871.0	F	5'- gtgagtaaggttaccgaattcCCGTCAGGAGACCTCAGTCTGG -3'
	R	5'- cgtgagctcggtaccggatccAACCTCTCCGATCCGAACGG -3'
Cluster-5693.0	F	5'- gtgagtaaggttaccgaattcTCCCCTGTCGTGATCACAATG -3'
	R	5'- cgtgagctcggtaccggatccACCCGTTCGGTGCGCCAG -3'
Cluster-5908.0	F	5'- gtgagtaaggttaccgaattcGCCGTCAGCTATGAGTCGGA -3'
	R	5'- cgtgagctcggtaccggatccTCATACGAGCGTCGAGGTTGT -3'
pTRV2	F	5’- ttacggacgagtggactag -3’
	R	5’- ctatggtaagacaatgagtcg -3’

Note: The primer sequence is written with small letters for the homologous arm on the expression vector.

Table S2: Primers used in qRT-PCR validation of candidate effector genes

Gene ID	Sequence（5'-3'）
Cluster-90573.0	F: 5'- AATGGTGGATGCCGTCAGAG -3'
	R: 5'- CTTTCGTGAGACCCGACACA -3'
Cluster-123950.0	F: 5'- ACTCCTACTGCCACCAATCC -3'
	R: 5'- ACGCTAGTAGGTGCTTGAGTG -3'
Cluster-116772.0	F:5'- TGTTGACCTCGTCGGAAACC -3'
	R: 5'- GCCGCTTCCAGTGAAGTAGT -3'
Cluster-9848.1	F: 5'- TGAACGTGCCAGTCAAGACC -3'
	R: 5'- AGGATGATTGGACCACCCGA -3'
Cluster-15140.0	F: 5'- TTCGCCAAGGTCAAGTCCAT -3'
	R: 5'- GAGAGCTGTAGGCGGTCTTG -3'
Cluster-117679.0	F: 5'- CTCTTACTCTGTCACTGGCGG -3'
	R: 5'- CATCCACGCCGGTAATGGTT -3'
Cluster-99695.0	F: 5'- TTGGGTGTTGACCTTCTCGG -3'
	R: 5'- GCGGCGGTGAAATACTTGTC -3'
Cluster-14572.0	F: 5'- CACGGACGAGTTTTCCCAGT -3'
	R: 5'- GTTCTCGTATGGGTGAGCGG -3'
Cluster-12918.0	F: 5'- GGCTACAAGAAGTCGTTCCCA -3'
	R: 5'- CTCAAGAGCATCACGAGCGG -3'
Cluster-15585.0	F: 5'- AGAGGCCAACTTCTCGACTC -3'
	R: 5'- CCACCGTTGCGATTTTGGTC -3'
Cluster-107849.0	F: 5'- ACCATTCAGAGCATCTCCACC -3'
	R: 5'- TGCTTGTCCGTCTACGATGA -3'
Cluster-93878.0	F: 5'- TTGCAGATTTGACAGCAACTGA -3'
	R: 5'- TTTTGAGGGAGTGGTGCATTT -3'
Cluster-13595.4	F: 5'- GGTATTGTGCTCGTCTCGCA -3'
	R: 5'- TAGTCACCCGTCACAATCGG -3'
Cluster-11592.0	F: 5'- GTCGGTGATGAACTCGTGGA -3'
	R: 5'- TTGCGTCCAGTAGTGCTTGT -3'
Cluster-10275.0	F: 5'- TGGTAGTGTGAGCAAGCTGT -3'
	R: 5'- CGTCCGTCGCATAAGTCAGA -3'
Cluster-64407.0	F: 5'- TACGAAAATGCGGCTAACGC -3'
	R: 5'- GATACCCTTTCTGCACGCGA -3'
Cluster-6477.0	F: 5'- TACATGCCAAAGGACTCGCC -3'
	R: 5'- CGACTTCTTGTAGCCGCTGT -3'
Cluster-123197.0	F: 5'- CACTTCATCTTCCCCGACCC -3'
	R: 5'- GGTGATGTGGTTGACCGAGT -3'
Cluster-28982.1	F: 5'- GTACCGTCACCATCGACCTG -3'
	R: 5'- CGCCGTAGTTGACGGTGTAG -3'
Cluster-19048.0	F: 5'- TCGATCGACATCTGCGGC -3'
	R: 5'- CCTGCTCGCTGTTCTTGGG -3'
Cluster-12482.0	F: 5'- CGCATCTACAGCCTCACAGA -3'
	R: 5'- GAGAGGAGCTGAGTGAACCG -3'
Cluster-108133.0	F: 5'- TGGGAACAGGTGCGAAATAGT -3'
	R: 5'- CCCTTATTGATCCTCCGCCA -3'
Cluster-11871.0	F: 5'- CGACGCGAGAAAAGACAGTT -3'
	R: 5'- CCGTGTTCTGTGTCTACGCC -3'
Cluster-5693.0	F: 5'- ATAGCCCTAGCCAATCGGTC -3'
	R: 5'- TCTGTATCTTGTGCAGCGGTT -3'
Cluster-5908.0	F: 5'- GGAGCCCTGCTTATCGAGTG -3'
	R: 5'- CGAGGTTGTCACTTGATGCC -3'

Table S3: Primers used in verification of the secretory function of signal peptide

Gene ID		Sequence（5'-3'）
	F	5'- cggaattttaattaagaattc -3'
	R	5'- cactatagggagaacctcgag -3'
Cluster-107849.0	F	5'- cggaattttaattaagaattcATGAAAATTGTGGTATTCCTTCTCAT-3'
	R	5'- cactatagggagaacctcgagATGAAAATTGTGGTATTCCTTCTCAT-3'
pSUC	F	5'- TTGTTTCCTCGTCATTGTTCTCG-3'
	R	5'- GGGTCATTCATCCAGCCCTTGTT-3'

Note: The primer sequence is written with small letters for the homologous arm on the expression vector..
